# cAMP Receptor Protein Controls Vibrio cholerae Gene Expression in Response to Host Colonization

**DOI:** 10.1128/mBio.00966-18

**Published:** 2018-07-10

**Authors:** Jainaba Manneh-Roussel, James R. J. Haycocks, Andrés Magán, Nicolas Perez-Soto, Kerstin Voelz, Andrew Camilli, Anne-Marie Krachler, David C. Grainger

**Affiliations:** a Institute of Microbiology and Infection, School of Biosciences, University of Birmingham, Edgbaston, Birmingham, United Kingdom; b Department of Molecular Biology and Microbiology, Tufts University, Boston, Massachusetts, USA; c Howard Hughes Medical Institute, Tufts University, Boston, Massachusetts, USA; d Department of Microbiology and Molecular Genetics, University of Texas McGovern Medical School at Houston, Houston, Texas, USA; National Cancer Institute

**Keywords:** *Vibrio*, biochemistry, gene regulation, genome analysis

## Abstract

The bacterium Vibrio cholerae is native to aquatic environments and can switch lifestyles to cause disease in humans. Lifestyle switching requires modulation of genetic systems for quorum sensing, intestinal colonization, and toxin production. Much of this regulation occurs at the level of gene expression and is controlled by transcription factors. In this work, we have mapped the binding of cAMP receptor protein (CRP) and RNA polymerase across the V. cholerae genome. We show that CRP is an integral component of the regulatory network that controls lifestyle switching. Focusing on a locus necessary for toxin transport, we demonstrate CRP-dependent regulation of gene expression in response to host colonization. Examination of further CRP-targeted genes reveals that this behavior is commonplace. Hence, CRP is a key regulator of many V. cholerae genes in response to lifestyle changes.

## IMPORTANCE

Cholera is an infectious disease that is caused by the bacterium Vibrio cholerae. Best known for causing disease in humans, the bacterium is most commonly found in aquatic ecosystems. Hence, humans acquire cholera following ingestion of food or water contaminated with V. cholerae. Transition between an aquatic environment and a human host triggers a lifestyle switch that involves reprogramming of V. cholerae gene expression patterns. This process is controlled by a network of transcription factors. In this paper, we show that the cAMP receptor protein (CRP) is a key regulator of V. cholerae gene expression in response to lifestyle changes.

## INTRODUCTION

Vibrio cholerae is a Gram-negative bacterium that causes the diarrheal disease cholera (1). Estimated to claim 3 to 5 million victims every year, cholera is endemic in regions of Asia and sub-Saharan Africa (1–4). Localized epidemics are also frequent; half a million cases have been attributed to the current outbreak in Yemen (2). Although notorious as a pathogen of humans, V. cholerae is native to aquatic environments (5). In this situation, the organism proliferates by colonizing crustaceans and other biota in their habitat (5–10). In particular, chitinous surfaces provide a substrate for biofilm formation and nutrients (5). Humans encounter V. cholerae following the ingestion of contaminated food or water (1). In response, the bacterium produces mucin-degrading enzymes and upregulates motility (5). This facilitates penetration of the intestinal mucosa (5). The subsequent attachment of V. cholerae cells to the intestinal epithelium requires toxin-coregulated pili (TCP) and accessory colonization factors (ACF) (11–13). Ultimately, disease results from the production of factors including cholera toxin (CTX), repeats in toxin (RTX), and hemolysin (HlyA) (42, 63–65).

Unsurprisingly, the expression of V. cholerae genes for quorum sensing, host colonization, and toxin production/export is precisely regulated (5). Most notably, an AraC/XylS family transcription factor called ToxT directly regulates the transcription of *ctxAB*, *acfAD*, and genes encoding the TCP (14). Production of ToxT is induced in the intestine and is codependent on two OmpR family regulators, ToxR and TcpP, which respond to extracellular signals that include osmolarity, pH, and bile (15–17). Genes encoding outer membrane porins OmpT and OmpU are also regulated by ToxR in a pathway that permits initial sensing of bile and subsequent resistance (18). Together, the aforementioned gene regulatory events comprise the ToxR regulon. Transcription factors with targets overlapping the ToxR regulon include VpsT, AphA, AphB, and the cyclic AMP (cAMP) receptor protein (CRP) (19–24). Best studied in Escherichia coli, CRP can activate transcription by binding targets centered either 41.5 or 61.5 bp upstream from a transcription start site (25). Since CRP binds DNA in response to the intracellular availability of cAMP, genes are controlled in response to nutrient availability (25–28). As such, CRP plays an integral role in the utilization of alternative carbon sources (29, 30). Hence, many E. coli genes that are differentially regulated in the intestine are controlled by CRP, including CTX-related toxins in pathogenic E. coli strains (26, 31). In V. cholerae, CRP is known to influence the ToxR regulon; CRP directly inhibits *tcpP* expression and activates the transcription of *ompT* (22, 32, 33). Remarkably, despite being a global regulator of transcription, direct control by CRP has only been demonstrated for seven V. cholerae genes (22, 31, 33–38). Furthermore, gene regulation by CRP during colonization of a host intestinal tract has never been studied. In this work, we have used chromatin immunoprecipitation (ChIP) coupled with DNA sequencing (ChIP-seq) to map the distribution of CRP across the V. cholerae genome. We show substantial overlap between the ToxR regulon and control of additional virulence factors not regulated by the ToxR system. Focusing on one such target, encoding RTX and its export system, we show that CRP is essential for specific induction of gene expression during intestinal colonization. Examination of additional CRP target genes reveals that similar effects are widespread.

## RESULTS

### Genome-wide distribution of CRP and RNA polymerase in Vibrio cholerae*.*

**FIG 1:**
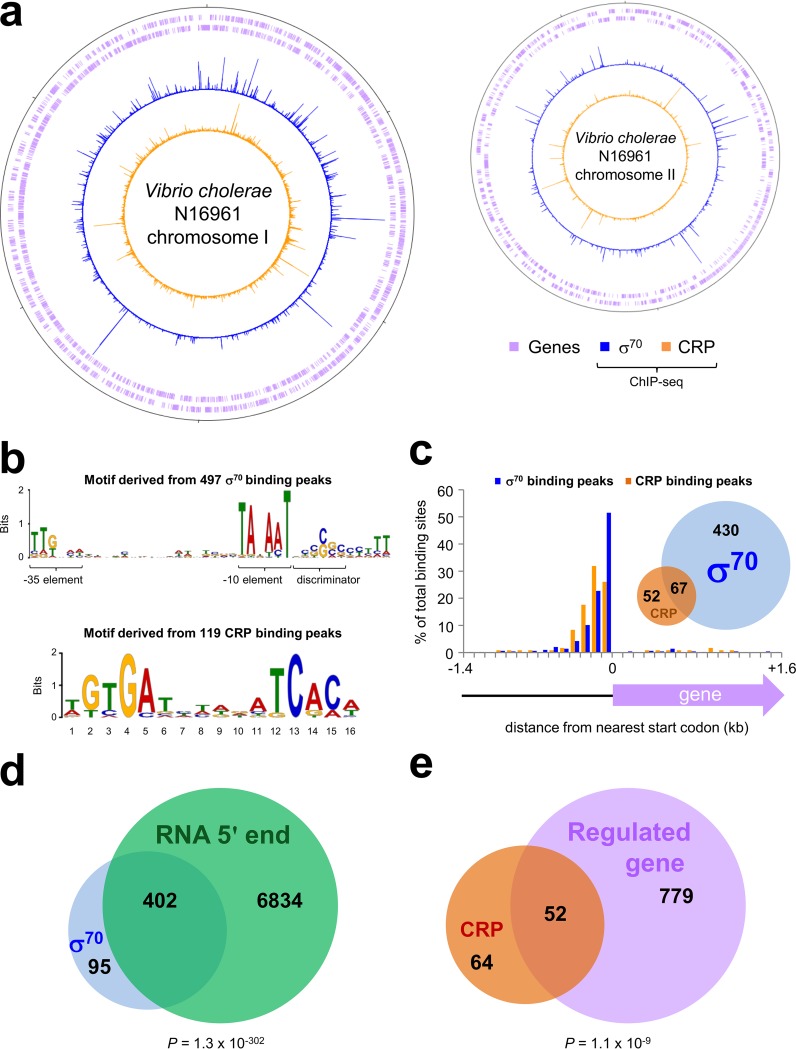
Global analysis of CRP and σ^70^ binding in Vibrio cholerae. (a) Genome-wide distribution of CRP and the RNA polymerase σ^70^ subunit in Vibrio cholerae strain N16961. Plots are shown for the two N16961 chromosomes. In each plot, the tick mark at the 12 o’clock position represents the first base pair (bp) of the chromosome and subsequent tick marks are spaced by 0.5 Mbp. In each plot, the first and second tracks (mauve lines) show the positions of genes, the third track (blue) is the σ^70^ binding profile, and the fourth track (orange) is the CRP binding profile. (b) DNA sequence motifs recovered from CRP and σ^70^ binding peaks. Top, DNA sequence motif identified by MEME present in DNA sequences associated with σ^70^ binding; bottom, DNA sequence motif generated from CRP binding peaks. (c) Locations of CRP and RNA polymerase binding peaks with respect to genes. Histogram depicts the distances between ChIP-seq binding peaks and the nearest 5′ end of a gene; data for CRP binding are in orange, and data for σ^70^ binding are in blue. Each binding peak was allocated to a series of 100-bp bins. Inset, Venn diagram that illustrates the number of overlapping CRP and σ^70^ binding peaks. (d) Overlap between σ^70^ DNA binding and transcription start sites. The Venn diagram illustrates numbers of overlapping σ^70^ binding sites (blue) and transcription start sites (green) (40). A σ^70^ binding peak centered within 50 bp of a transcription start was considered to overlap. To generate the *P* value, we used the chi-square test. To generate values for the expected overlap between the data sets, assuming no correlation, we randomized the positions of the σ^70^ peaks. (e) Overlap between CRP binding and CRP-regulated genes. The Venn diagram illustrates overlap between genes adjacent to CRP binding peaks (orange) and genes that were differently expressed in the absence of CRP (mauve) (41). To generate the *P* value, we used the chi-square test. To generate values for the expected overlap between the data sets, assuming no correlation, we randomly selected 831 genes from the V. cholerae genome and determined the number that were adjacent to CRP binding peaks.

We used ChIP-seq to map global DNA binding by CRP and the RNA polymerase σ^70^ subunit in V. cholerae strain N16961 grown to mid-log phase in M9 minimal medium supplemented with 1% (wt/vol) fructose (39). The strain, isolated from a Bangladeshi patient in 1971, comprises 3,885 genes borne on two circular chromosomes of 2,961,146 bp (chromosome I) and 1,072,314 bp (chromosome II) (39). The binding profiles of CRP and σ^70^ are shown in Fig. 1a. In each plot, genes are illustrated by mauve lines (Fig. 1a, first and second tracks), σ^70^ binding in blue (Fig. 1a, third track), and CRP binding in orange (Fig. 1a, fourth track). We identified 497 binding peaks for σ^70^ and 119 binding peaks for CRP. The σ^70^ peaks were not distributed equitably; chromosome II accounts for 27% of the V. cholerae genome but aligned with 40% of the σ^70^ binding peaks. To assess the validity of our data, we examined the DNA sequence attributes of each peak. Hence, we used MEME (Multiple Em for Motif Elicitation) to identify sequence motifs associated with σ^70^ or CRP binding. The most statistically significant DNA motif associated with each group of peaks is shown in Fig. 1b. As expected, MEME recovered significant motifs matching the sequence of a housekeeping bacterial promoter (*E* = 4.5 × 10^−29^) (Fig. 1b, top) and the palindromic CRP binding sequence (*E* = 2.5 × 10^−2^) (Fig. 1b, bottom). For all peaks, we determined the distance to the nearest start codon and sorted these distances into 100-bp bins. The distribution of peaks among the bins is illustrated in Fig. 1c; σ^70^ most frequently binds the 100 bp preceding the 5′ end of a gene, while CRP binds further upstream. Of the 119 CRP binding peaks, 67 colocated with binding of σ^70^ (Fig. 1c, inset). We also compared our data with existing compendiums of the V. cholerae transcriptome (40, 41). Briefly, Papenfort and coworkers used differential RNA-seq to map transcription start sites (TSS) in V. cholerae (40). There was significant overlap with our data; 81% of the σ^70^ binding peaks matched a TSS (*P* = 1.3 × 10^−302^) (Fig. 1d). Thus, the combined data describe sigma factor preference, promoter sequence, and sites of transcription initiation for the majority of V. cholerae transcription units. In a separate study, Fong and Yildiz used DNA microarrays to detect changes in RNA levels resulting from *crp* deletion (41). Again, the overlap was significant, and 52 of the 119 CRP binding peaks identified a differentially expressed gene (*P* = 2.1 × 10^−9^) (Fig. 1e). Note that greater overlap of the CRP binding and gene regulatory data is not expected; many CRP-controlled promoters are active only under specific conditions, and most transcriptome changes will result from indirect effects of CRP (26–30).

### Expression of the *rtxBDE* operon is repressed by CRP.

**FIG 2:**
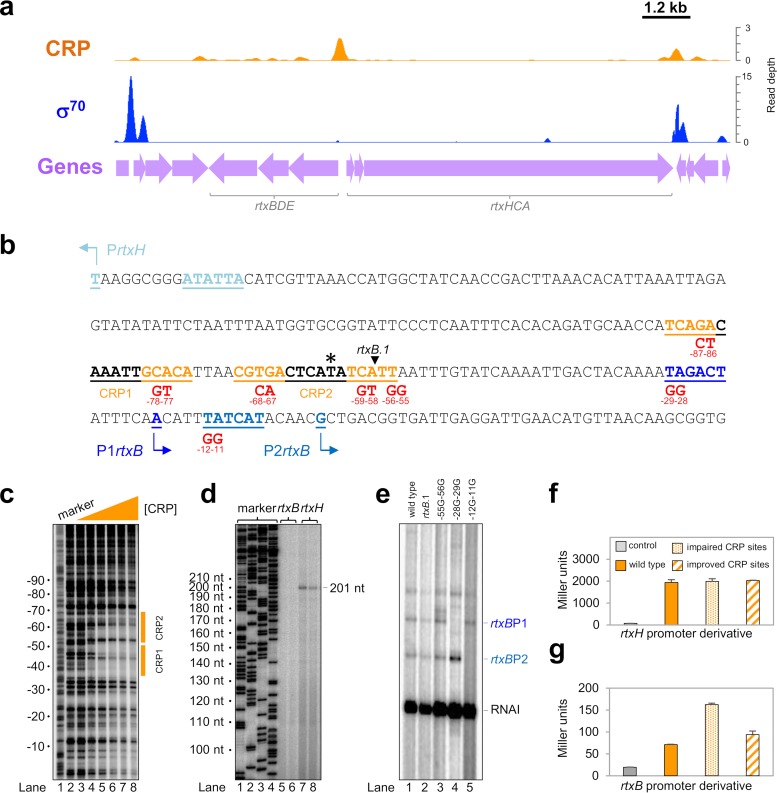
Repression of the *rtxBDE* operon by CRP. (a) The intergenic region between *rtxBDE* and *rtxHCA* is associated with CRP but not σ^70^. The graphs illustrate ChIP-seq data for CRP (orange) and σ^70^ (blue) binding to the *rtx* locus. Data have been smoothed in a 100-bp window. Genes are depicted by mauve arrows and labeled. (b) Sequence of the *rtxBDE-rtxHCA* gene regulatory region. The DNA sequence between *rtxBDE* and *rtxHCA* is shown. The center of the CRP binding peak identified in our ChIP-seq analysis is indicated by an asterisk. Putative CRP sites (orange) are underlined and labeled. The *rtxH* transcription start site (+1) is underlined and further highlighted by a bent arrow. The associated promoter −10 element is similarly colored and underlined. Two transcription start sites for *rtxB* are also labeled in the same way. The 5′ end of the *rtxB*.1 DNA fragment (see the legend to panel e) is indicated by an inverted black triangle. Point mutations used to inactivate CRP binding sites or promoter −10 elements are shown in red. (c) DNase I footprint of CRP binding to the *rtxBDE-rtxHCA* gene regulatory region. Results of a DNase I footprinting experiment using the *rtxBDE* intergenic region and purified V. cholerae CRP. The experiment is calibrated with a Maxam-Gilbert GA sequencing ladder, and positions relative to the P1*rtxB* transcription start site (+1) are labeled. The triangle indicates the addition of CRP at concentrations of 175, 350, 700, 1,400, 2,100, or 2,800 nM. The positions of the predicted CRP binding sites are shown by orange boxes. (d) Primer extension analysis of *rtxH* and *rtxB* promoter-derived transcripts. The gel shows arbitrary Sanger sequencing reactions for calibration (lanes 1 to 4) and primer extension products for *rtxB* (lanes 5 and 6) or *rtxH* (lanes 7 and 8) promoter-derived transcripts. (e) Transcripts derived from the *rtxBDE* intergenic region *in vitro*. The gel shows transcripts generated by V. cholerae RNA polymerase σ^70^ holoenzyme using the *rtxBDE* intergenic region, cloned in plasmid pSR, as a DNA template. The RNAI transcript is derived from the plasmid replication origin and serves as an internal control. The *rtxB*.1 derivative contains a truncated version of the *rtxBDE* intergenic region. The site of the truncation is marked in panel b. Mutations introduced to disrupt potential −10 hexamers are noted above the gel and are also shown in panel b. (f) Activity of P*rtxH* is not affected by CRP. Results of a β-galactosidase assay done using lysates of N16961 cells transformed with derivatives of the *lacZ* reporter plasmid, pRW50T, where *lacZ* expression is controlled by P*rtxH*. (g) Expression of *rtxB* is repressed by CRP. Results of a β-galactosidase assay done using lysates of N16961 cells transformed with derivatives of the *lacZ* reporter plasmid, pRW50T, where *lacZ* expression is controlled by P1*rtxB* and P2*rtxB*.

Many V. cholerae genes involved in pathogenicity were targeted by CRP (Table 1). We focused our attention on the gene cluster responsible for the cytotoxic activity of V. cholerae (42). The region comprises two operons called *rtxHCA* and *rtxBDE*. The *rtxA* gene encodes RTX toxin, while *rtxH* and *rtxC* encode a hypothetical protein and an acyltransferase, respectively. The divergent *rtxBDE* operon encodes components of the toxin secretion system. Figure 2a shows binding of CRP and σ^70^ to DNA between the two operons. The sequence of the intergenic region is shown in Fig. 2b. Two putative CRP sites (orange and underlined) overlap the center of the CRP ChIP-seq binding peak (Fig. 2b, asterisk). To confirm binding, we purified V. cholerae CRP for use in DNase I footprinting assays. The footprinting data are consistent with CRP binding to both of the putative sites (Fig. 2c). To identify promoters of *rtxHCA* and *rtxBDE* transcription, we examined our ChIP-seq data for σ^70^ and the TSS mapping data of Papenfort et al. (40). However, these were poorly informative; the σ^70^ binding levels were low and Papenfort et al. identified only a single intergenic promoter for *rtxHCA*. Indeed, the TSS data suggest that the region is prone to spurious intragenic transcription; the *rtxHCA* and *rtxBDE* genes contain a total of 21 internal promoters (40). To map canonical promoters in the *rtxHCA*-*rtxBDE* intergenic region, we used two approaches; mRNA primer extension and *in vitro* transcription. The results of the primer extension analysis are shown in Fig. 2d. We were unable to derive any extension products from *rtxBDE* transcripts, but a single 201-nucleotide extension product was generated from the *rtxHCA* transcript. The corresponding TSS aligns perfectly with the *rtxHCA* TSS identified by Papenfort et al. (labeled P*rtxH* in Fig. 2b) (40). A promoter −10 element is appropriately positioned upstream, and deletion of this sequence abolishes mRNA production (see Fig. S1a in the supplemental material). Since both RNA-seq and primer extension failed to identify promoters for *rtxBDE*, we reasoned that the operon must be repressed *in vivo*. Hence, the *rtxBDE* intergenic region was cloned upstream from the λ*oop* terminator in plasmid pSR to create a template for *in vitro* transcription. The result of the experiment is shown in lane 1 of Fig. 2e. The control RNAI transcript is derived from the plasmid replication origin, and further transcripts could originate within the cloned intergenic DNA. Truncation of the *rtxBDE* intergenic region did not prevent synthesis of the additional RNAs (Fig. 2e, lane 2). Hence, the transcripts originate downstream from the truncation site marked by the inverted triangle in Fig. 2b. We made derivatives of the truncated DNA template with point mutations in all potential −10 hexamers. The mutations are illustrated in Fig. 2b. Two pairs of mutations, −29G −28G and −12G −11G, each prevent the production of a different transcript (Fig. 2e, lanes 3 to 5). The mutations have similar effects *in vivo* (Fig. S1b). We conclude that transcription originates from the promoters labeled P1*rtxB* and P2*rtxB* in Fig. 2b. Neither CRP site is appropriately positioned to activate P*rtxH*, P1*rtxB*, or P2*rtxB*. However, it has previously been shown that pairs of CRP binding sites upstream from promoters can repress transcription (43). Thus, we created derivatives of the intergenic region where the CRP sites were inactivated by the point mutations shown in Fig. 2b. We also altered the sites to match the consensus for CRP binding. The various DNA fragments were cloned in the appropriate orientation upstream from *lacZ* in plasmid pRW50T. The resulting DNA constructs were moved into V. cholerae strain N16961 by conjugation. Promoter activity was inferred by measuring β-galactosidase activity in lysates of the exconjugants. The data show that P*rtxH* activity is unaltered by any of the mutations (Fig. 2f). Conversely, the poorly active *rtxBDE* promoters have higher levels of activity when the CRP sites are mutated (Fig. 2g, stippled bar). Consistent with our observations, Fong and Yildiz reported repression of *rtxBDE* by CRP in their transcriptome analysis (41).

10.1128/mBio.00966-18.2 FIG S1  (a) Transcription from P*rtxH* requires identified promoter elements. (i) The results of primer extension assays that detect the *rtxH* transcript derived from plasmid pRW50T carrying the *rtxH* (panel ii, top) or truncated *rtxH*.1 (panel ii, bottom) DNA fragment. (ii) P*rtxH* is highlighted blue and CRP binding sites are shown in orange. (b)** **P1*rtxB* and P2*rtxB* make similar contributions to *rtxB* transcription. The figure shows β-galactosidase activity measurements for lysates of V. cholerae cells transformed with pRW50T derivatives carrying the full-length *rtxB* regulatory region (*rtxB*), a truncated derivative lacking CRP binding sites (*rtxB*.1), or versions of the truncated fragment with indicated promoter mutations. (c) Effects of *crp* and *tcpA* on zebrafish larva colonization. (i) Images from multiple zebrafish larvae colonized with the indicated V. cholerae strains. All strains were transformed with plasmid pMW-GFP to facilitate visualization of bacteria. (ii) Quantified fluorescence from multiple microscopy images. (d) Raw gel images. Download FIG S1, PDF file, 0.4 MB. Copyright © 2018 Manneh-Roussel et al. 2018 Manneh-Roussel et al. This content is distributed under the terms of the Creative Commons Attribution 4.0 International license.

**TABLE 1:** CRP binding peaks identified by ChIP-seq in the V. cholerae strain N16961 genome

Genome component	Peak center^a^	Site center^b^	Site *P* value^c^	Sequence^d^	Gene(s) close to CRP binding sites^e^	CRP regulated^f^
Chromosome I	55771	55796.5	3.5E−03	AGTGACTAAGCGTACA	23Sa1	ND
	99871	99790.5	8.3E−03	TGTTACGAATATTACA	*glpE*<(VC0103)	Yes
	134815	134755.5	5.6E−03	TTTGTTTTGGATCGAT	VC0142a<>VC0143	Yes
	150732	150753.5	3.2E−07	TGAGATTCAAATCACA	VC0159<>16Sb	Yes
	177320	177327.5	3.2E−03	CATAATCTGTATCAAA	VC0175	Yes
	242521	242505.5	7.1E−03	TAAGGTTTAAGCCATT	(VC0237)	No
	249227	249187.5	9.8E−03	TTTGAAGGATGGCGTT	(VC0242)	No
	264229	264210.5	5.1E−03	TTAAATATGTATCATA	VC0258><VC0259	No
	274582	NA^g^	NA	NA	(VC0269)	No
	294153	294127.5	7.6E−03	TGTAGGTGATATCTCA	VC0284	No
	454607	454517.5	3.4E−03	TGTGTTGTTGCTCAAT	VC0423<>VC0424	Yes
	516492	516436.5	1.7E−03	TGACAGTAATATCACT	VC0485<>VC0486	No
	522621	522571.5	5.3E−03	AGTCCATTTGCTCACA	VC0489	No
	527963	527988.5	1.1E−03	AGTTATTTTTTTCACT	VC0493<>VC0494	No
	569841	569895.5	4.9E−04	AACGATTTTCCTCATA	VC0537<>VC0538	Yes
	657711	657766.5	5.2E−05	TGTGACTCCCTTCGCA	VC0621	No
	676093	676049.5	2.4E−03	AATGATATAAATCCAA	*ompU*<>*greA*	Yes
	707829	707908.5	7.3E−03	GCCGCTTGGCATCACA	VC0661<>VC0662	No
	712256	712206.5	1.6E−03	TGCAATCTAAGTCATT	VC0665	No
	713923	714007.5	1.1E−03	TTAGAATTTAATCGTA	VC0666	No
	748263	748338.5	4.1E−03	GGCGAGATTACGCGTA	VC0699<>VC0700	No
	756779	756854.5	8.4E−07	TGTGATAAAAGTCACT	VC0706	No
	762509	762522.5	1.8E−03	TCTGACAATTATCTCG	VC0713	Yes
	788515	788501.5	6.9E−03	TGTGAAATTTCACAAG	VC0734	No
	815345	815346.5	3.6E−07	TGTGATATGATTCACA	*engA*	Yes
	818079	818158.5	2.6E−03	GGTTAATTAAGTCGCA	VC0765	Yes
	819917	819936.5	1.0E−02	CGTCCGCAATATCAAA	VC0766<>VC0767	No
	880295	880357.5	5.3E−03	TATGAGAAAGATAAAA	(VC0821)	No
	888697	888746.5	2.1E−03	TGCAATTAAGTTCTCA	*tcpI*<>*tcpP*	Yes
	894874	894817.5	7.9E−03	TATTATTGGATTCATT	(VC0833)	No
	904634	NA	NA	NA	(VC0842)	Yes
	906219	NA	NA	NA	*acfA*<>*acfD*	No
	911066	911128.5	3.1E−03	TATGATGAAAAACATT	VC0845><VC0846	No
	936058	936026.5	1.5E−03	AAAGAGCTAAATCGTT	(VC0870)	No
	999018	999093.5	9.6E−03	CTTGGTTGTTTTCAAT	VC0932<>VC0934	Yes
	1011835	1011745.5	2.4E−03	AGTGAGCTTGCCCAAG	(VC0947)	No
	1037560	1037566.5	1.2E−03	TTCGACGCATTTCAAA	VC0972	Yes
	1054013	1054031.5	1.4E−05	CGTGATTTTTGTCGCG	*tppB*<>*rfaH*	No
	1061159	1061198.5	1.5E−06	GGTGATTAGGATCACA	*nagA*<>VC0995	No
	1090145	1090112.5	5.6E−04	TGTGATGTTTGGCATC	VC1021	No
	1100204	1100264.5	4.0E−05	TGTGATGCAAATCGAT	VC1034	Yes
	1139471	1139535.5	4.0E−03	TCTGATTATTTTCAAG	VC1073	No
	1174954	1174946.5	6.7E−06	TGTGGTTTATGTCACA	VC1104	No
	1198758	1198847.5	6.0E−05	TGTGAGCTGTGGCACT	VC1130<>VC1131	No
	1212539	1212568.5	4.7E−03	AGAGGCGAAATTCATT	VC1142<>*clpS*	Yes
	1224786	1224787.5	4.2E−06	TGTGATACTGGTCTCA	VC1152<>*tfoX*	No
	1382925	1382894.5	5.9E−03	TGTGAGAATTGTTAAT	VC1301	Yes
	1396772	1396717.5	1.2E−03	ATTGATGTCACTCAAA	VC1313<>VC1314	Yes
	1408936	1408937.5	3.8E−03	TTTTAACTGGTTCACA	VC1323<>VC1325	Yes
	1549042	1549038.5	6.9E−03	TGTGCAATTTGTCTGA	*rtxB*<>*rtxH*	Yes
	1568164	1568072.5	5.5E−03	TATGAAAATGATGATA	*ctxA*	No
	1683652	1683636.5	2.0E−03	AGTGATGGGGTTAACA	VC1571<>VC1572	No
	1703584	1703620.5	6.4E−03	TAATAAAAATGTCACA	VC1592	No
	1741600	1741668.5	4.7E−05	TGTGATACGCTTCTCG	VC1620<>VC1621	Yes
	1776678	1776642.5	4.8E−03	AGTGATTTATCACTAA	VC1649<>VC1650	No
	1789510	1789532.5	1.9E−05	TATGACCAGTATCGCA	VC1656<>VC1658	No
	1903470	1903498.5	8.0E−04	TTTGAGTTAATTCAAT	(VC1736)	Yes
	1919651	1919579.5	6.2E−03	TGTGCTAAATACAACG	(VC1771)	Yes
	1922932	NA	NA	NA	(VC1773)	No
	1967295	1967278.5	2.1E−06	CGAGATCTAAATCACA	VC1825<>VC1826	No
	1984776	1984846.5	3.3E−04	TGAGAACTTTGTCAAA	VC1844	Yes
	1990074	1990055.5	4.4E−03	GTCGAGACCACTCATA	VC1851	No
	1994054	1994088.5	5.4E−03	ATTAATAAAAATCAAA	*ompT*<>*dinG*	Yes
	2004839	2004771.5	2.9E−03	TTTTAACAAAGTCACA	VC1864<>VC1865	Yes
	2055395	2055442.5	6.2E−03	CATCAAATTTTTCACA	VC1904<>VC1905	Yes
	2059077	2059085.5	9.7E−03	TGCCACGCAACGCTCA	*cysB*<>VC1909	Yes
	2168387	2168407.5	4.2E−03	TTTGAGGAATTCCGCT	VC2013	Yes
	2190666	2190734.5	5.0E−03	TGTGCGAATGTTAACA	VC2035	No
	2193110	2193078.5	1.1E−05	AACGATATAAATCACA	VC2036<>VC2037	Yes
	2374476	2374498.5	3.0E−05	TGTGAGCTTTATCATG	VC2219<>VC2220	No
	2433743	2433742.5	1.1E−06	GGTGATTAAAATCACA	VC2278<>VC2279	No
	2457608	2457631.5	1.3E−06	AGCGATTAAGATCACA	VC2303<>VC2305	No
	2537389	2537396.5	4.6E−03	TGTGAATTCGGTGAAA	*gltB*	No
	2550352	2550386.5	8.7E−03	TGTTACTGGTATAACA	(VC2385)	No
	2551356	2551299.5	3.5E−03	AGTGATAAAAGTGAAG	(VC2386)	No
	2558608	2558615.5	3.7E−03	GATGAATTTATTCATC	VC2390	Yes
	2610382	2610307.5	9.8E−03	GCTGATTCGCGTCTTG	VC2435<>*tolC*	No
	2653838	2653780.5	7.5E−04	CGCGAGTCTCTTCAAA	VC2473	Yes
	2667326	2667406.5	8.8E−03	TAATATTCACGTCAAA	VC2486	No
	2699390	2699329.5	1.5E−03	GGTGATGGTCGCCACT	*pyrB*	No
	2743349	2743361.5	8.1E−04	ATCGCGTCACATCACA	VC2561<>*cpdB*	No
	2787939	2787903.5	3.0E−04	TGAGATAAACCCCACA	VC2618	Yes
	2845246	2845280.5	5.7E−07	TGTGATTTTCATCACG	VC2677	No
	2864757	2864763.5	9.2E−04	ATAGATAAAACTCTCA	VC2698<>*aspA*	Yes
	2933468	2933432.5	7.5E−04	TTTGATTATCATCAAC	16sg	ND
	2936869	2936904.5	3.1E−04	TTCGATACCAAGCACA	23Sh	ND

Chromosome II	12067	12085.5	5.4E−08	TGTGATCCGAATCACT	VCA0012<>VCA0013	Yes
	86364	86274.5	5.8E−03	GTCGAAATTCGCCACA	VCA0076	No
	99016	98927.5	2.0E−07	TGTGATCTTTATCACT	VCA0089	No
	114856	114864.5	8.6E−03	TTTAATAGATTTCTCA	VCA0104<>VCA0105	No
	152867	152849.5	7.4E−04	TGTGATTGATGTGGCA	VCA0138	No
	181749	181688.5	2.5E−03	TGAGAAAGCATTCAAA	VCA0164<>VCA0165	No
	217815	217798.5	6.0E−03	TGTTATAAAAACCAAT	(VCA0200)	No
	237015	237049.5	6.6E−03	TAAGAATTATTTTACA	*hlyB*<>*hlyA*	No
	247246	247185.5	7.5E−03	TTGGCATAGCATCACA	VCA0224<>VCA0225	Yes
	267292	267253.5	3.7E−03	TGATAGGTAGATCACC	VCA0246<>VCA0247	No
	300413	300391.5	8.4E−03	TGCCCTATCTATCAAA	VCA0281	Yes
	334916	334914.5	4.3E−03	ATTGACAGCTATCTAA	(VCA0334)	No
	458259	458254.5	3.8E−03	CGTGATTAAAAACGTC	VCA0523	Yes
	481906	481918.5	2.0E−03	TTTCATAAAAGTCACG	VCA0544<>VCA0545	Yes
	492167	492243.5	6.9E−07	TGTGATTGGAATCACT	VCA0554<>VCA0556	No
	564616	NA	NA	NA	(VCA0628)	Yes
	598381	598370.5	4.9E−04	GTTGACAACAGTCACA	(VCA0662)<>VCA0663	No
	630430	630499.5	1.5E−03	AATGATAGATAACACA	VCA0691	Yes
	687485	687472.5	3.3E−03	CGTGATCGACATTAAA	(VCA0742)>VCA0743	No
	741821	741822.5	7.8E−06	TGTGCTTTACATCACT	VCA0801	Yes
	784413	784364.5	2.1E−05	TGTGATGCCGCTCGCA	VCA0840	Yes
	785337	785352.5	5.1E−04	TTTGAACTTAGTCATT	VCA0843	Yes
	801056	801043.5	1.7E−05	TGTGAAATGGCTCGCA	VCA0849	Yes
	832501	832514.5	6.2E−03	TGCGACCTTGATTAAC	VCA0880	Yes
	849892	849906.5	3.0E−03	GTTGACGCCTTTCTCA	VCA0896	Yes
	870862	870876.5	1.1E−03	AATGATCAGGGGCAAA	VCA0917<>VCA0919	Yes
	874452	874411.5	4.1E−03	TATAAATCAAATCATT	VCA0923	Yes
	897315	897352.5	4.8E−06	AGCGAGCCAAATCACA	VCA0945<>VCA0946	Yes
	902411	902377.5	6.6E−03	TGAAACACTTACCACT	VCA0952	Yes
	930851	NA	NA	NA	(VCA0982)<>VCA0983	No
	963517	963536.5	8.2E−04	TGTTAAGCAAATCGCA	VCA1013<>VCA1015	No
	994614	994588.5	2.5E−05	CATGACACAGGTCACA	VCA1043<>(VCA1044)	Yes
	1015957	1015902.5	2.0E−05	TTTGACCATTATCACA	VCA1063	No

a Center of peak for CRP binding in ChIP-seq assays.

b Center of binding site identified by FIMO (Find Individual Motif Occurrences) using DNA motif recovered from the ChIP-seq data by MEME (Multiple Em for Motif Elicitation).

c *P* value assigned to each site by FIMO describing the significance of the match to the motif generated by MEME.

d DNA sequence of site identified by FIMO.

e Parentheses indicate that the CRP site is located within that gene. Pairs of arrows represent divergent (<>) or convergent (><) genes. Single arrows indicate that gene pairs are in the same orientation on either the forward (>) or reverse (<) strand. Gene identification numbers are shown unless an alternative name for the gene is provided in the genome annotation or the wider literature. Genes regulated by ToxR or ToxT are underlined.

f CRP-regulated genes described by Fong and Yildiz (41). ND, not detected: genes encoding stable rRNA species were not included in the transcriptome analysis and so no change in transcription could be detected.

g NA, not applicable.

### Expression of the *rtxBDE* operon responds to nutrient availability in a CRP-dependent manner.

**FIG 3:**
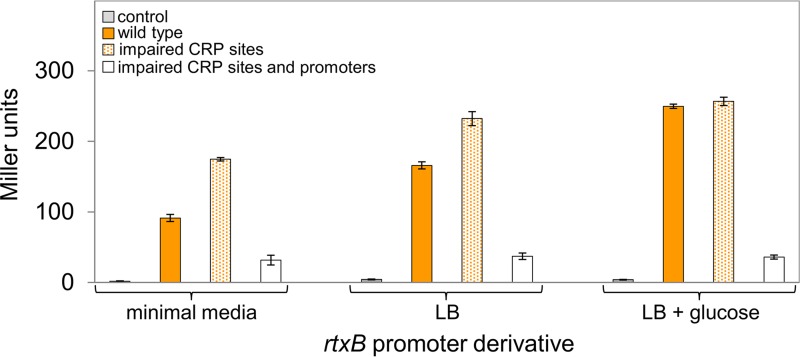
Nutrient availability controls *rtxBDE* expression in a CRP-dependent manner. Results of β-galactosidase assays done using lysates of N16961 cells transformed with derivatives of the *lacZ* reporter plasmid, pRW50T, carrying different *rtxB*::*lacZ* fusions. Cells were grown in M9 minimal medium, LB, or LB supplemented with 0.4% (vol/vol) glucose.

The ability of CRP to bind DNA *in vivo* is regulated by nutrient availability. Hence, CRP binds to target sites when cells are grown in M9 minimal medium but binding is reduced in lysogeny broth (LB) and abolished upon the addition of glucose (25–28). As such, repression of *rtxBDE* should be relieved in rich medium. To test this, we used strain N16961 carrying the *rtxB*::*lacZ* fusions on pRW50T. The various strains were grown in M9 minimal medium, LB broth, or LB broth supplemented with 0.4% glucose. As expected, β-galactosidase activity due to the *rtxB*::*lacZ* fusion increased in LB broth and rose further upon the addition of glucose (Fig. 3, compare solid orange bars). Furthermore, inactivation of the CRP sites had a reduced effect in LB broth and no effect when glucose was present (Fig. 3, compare solid and stippled orange bars for each growth condition). Importantly, the sizes of changes in gene expression observed were similar to data for other CRP-regulated promoters (31). We confirmed that the observed gene expression was due to P1*rtxB* and P2*rtxB*. Hence, mutation of the promoter −10 elements greatly reduced *lacZ* expression (Fig. 3, open bars).

### CRP plays an important role during colonization of an aquatic host.

**FIG 4:**
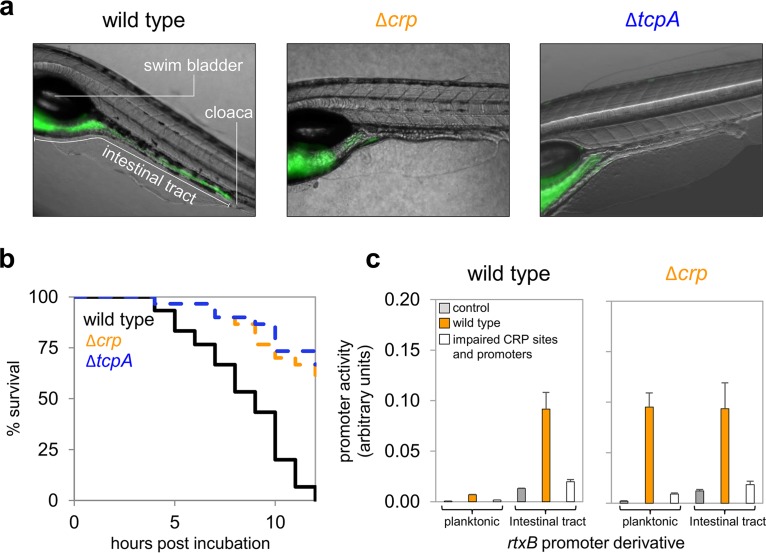
CRP is required for efficient host colonization and dependent induction of *rtxBDE*. (a) Colonization of the zebrafish larva intestinal tract by V. cholerae strain E7946 and derivatives. The three panels show representative fluorescence microscopy images overlaid on light microscopy images of zebrafish larvae colonized with the indicated V. cholerae strains. All bacterial strains were transformed with plasmid pMW-GFP and express green fluorescent protein (GFP) to facilitate detection. Further images are shown in Fig. S1c in the supplemental material. (b) Survival of zebrafish larvae following infection with V. cholerae strain E7946 and derivatives. (c) Expression of *rtxBDE* is induced by zebrafish larva colonization. Results of a β-galactosidase assay done using lysates of bacterial cells growing planktonically in E3 medium or obtained from the zebrafish intestinal tract. Strains are indicated and were transformed with derivatives of pRW50T encoding different *rtxB*::*lacZ* fusions.

Although largely overlooked as a vector for V. cholerae, a recent study found that up to 87% of fish species were colonized by the bacterium in certain localities (7). Indeed, it has been suggested that colonization of fish has sustained the epidemicity of cholera in India (8). Since fish and humans have similar gut mucosa, the former have emerged as a model to study intestinal colonization (9, 10). The zebrafish larva model is particularly useful; bacteria are added to saltwater solutions in which larvae are free swimming and colonization follows without intervention (10). Given that changes in nutrient availability are associated with host colonization, we examined the role of CRP in this process. Figure 4a shows representative images of zebrafish larvae infected with V. cholerae strain E7946 or derivatives. Further images for each strain are shown in Fig. S1c. All strains express green fluorescent protein to facilitate their visualization. Infections due to the wild-type strain are disseminated throughout the intestinal tract (Fig. 4a, left). Conversely, infections caused by cells lacking CRP or TCP are limited to the upper intestinal tract and fail to colonize the midintestine and posterior intestine (Fig. 4a, middle and right). Quantification of fluorescence in microscopy images revealed 3-fold reductions for the Δ*crp* and Δ*tcpA* strains relative to the amount in the wild type (Fig. S1c). We also monitored survival of the larvae during incubation with the bacteria (Fig. 4b). All larvae infected with wild-type V. cholerae were dead by the end of the time course (Fig. 4b, black line). Conversely, infections caused by strains lacking CRP or TCP were not usually fatal (Fig. 4b, orange and blue lines). Hence, both CRP and TCP are important for colonization of fish (22).

### Induction of the *rtxBDE* operon during host colonization is mediated by CRP.

We next considered the possibility that transcription from the *rtxBDE* promoters might be triggered during colonization of the intestinal tract. To test this, zebrafish larvae were colonized with either wild-type or Δ*crp* derivatives of V. cholerae carrying *rtxB*::*lacZ* fusions in plasmid pRW50T. After colonization, planktonic bacteria were recovered from the water and larvae were sacrificed to release the intestinal bacteria. The levels of β-galactosidase expression were then determined from lysates of the two populations. The data obtained for wild-type V. cholerae are shown in the left panel of Fig. 4c. Low *rtxBDE* expression was measured for planktonic V. cholerae. However, *rtxBDE* expression increased substantially during colonization of the larval intestinal tract. As expected, this increase in expression required P1*rtxB* and P2*rtxB* (Fig. 4c, compare orange and open bars). In cells lacking CRP, the expression of *rtxBDE* was uncoupled from host colonization. Hence, high *rtxBDE* expression was measured in planktonic as well as intestinal populations (Fig. 4c, right). Note that the differences in gene expression observed are not due to the different colonization properties of the Δ*crp* strain; deregulation of *rtxBDE* occurs in planktonic populations rather than within the larvae. Furthermore, any differences in bacterial cell numbers were accounted for by normalization.

### CRP modulates the expression of many V. cholerae genes during host colonization.

**FIG 5:**
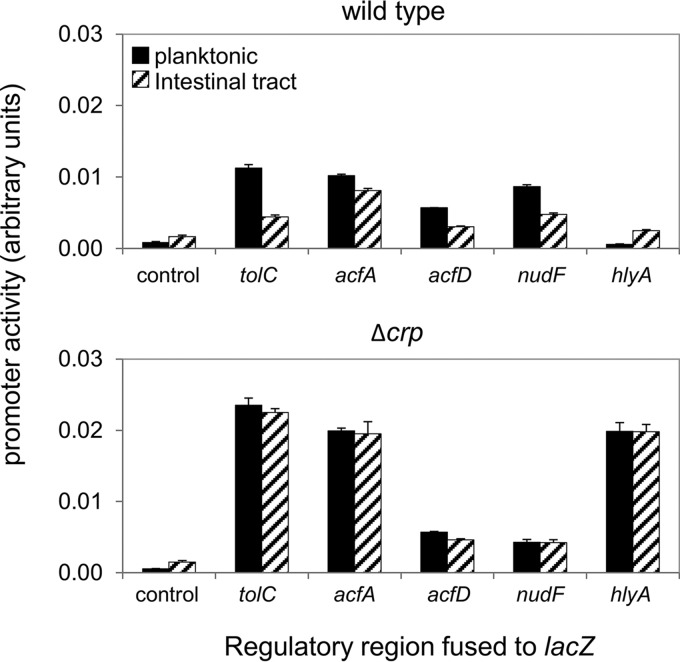
CRP couples the expression of many V. cholerae genes to host colonization. Results of β-galactosidase assays done using lysates of bacterial cells grown planktonically in E3 medium or obtained from the zebrafish intestinal tract. Strains are indicated and were transformed with derivatives of pRW50T encoding different LacZ fusions. Significant differences between levels of β-galactosidase activity in planktonic and intestinal populations were observed in all cases for wild-type cells (*P* values determined using a two-tailed Student’s *t* test were 0.0014, 0.0011, 0.0003, 0.0005, and 0.0037 for the *tolC*, *acfA*, *acfD*, *nudF*, and *hlyA* promoters, respectively). For cells lacking CRP, a significant, albeit much smaller difference was only apparent for the *acfD* promoter (*P* = 0.0036).

We reasoned that other CRP-targeted promoters would lose the ability to differentiate between aquatic environments and the host intestinal tract when CRP was absent. To test this, the promoters of the following five genes were selected, using our ChIP-seq data as a guide: *tolC* (encoding an outer membrane channel important for bile tolerance), *acfA* (encoding accessory colonization factor A), *acfD* (encoding accessory colonization factor D), *nudF* (encoding a pyrophosphatase), and *hlyA* (encoding hemolysin) (44). The promoter region of each gene was cloned upstream from *lacZ* in plasmid pRW50T, and the β-galactosidase activity was determined (Fig. 5a). The data show that all of the genes were expressed at different levels in planktonic (solid bars) and intestinal (striped bars) populations. The experiment was repeated in cells lacking Δ*crp* (Fig. 5b). The expression of all genes was rendered insensitive to host colonization (Fig. 5b, compare solid and striped bars).

## DISCUSSION

The ability of V. cholerae to persist in environmental reservoirs, colonize the intestinal tract, and cause disease requires careful coordination of gene expression (5). This process is best characterized for key virulence factors that collectively reside in the ToxR regulon (5, 12, 14). In this paper, we have investigated the role of CRP. We show that CRP targets five of the nine ToxR regulon transcription units. Hence, we identified binding sites for CRP adjacent to *ompU*, *acfA*, and *acfD*, in addition to the known targets *ompT* and *tcpPH*. Other genes involved in V. cholerae pathogenicity were also targets for CRP. These included *rtxBDE*, *tolC*, and *hlyA*. Previous transcriptome analysis led to speculation that CRP may modulate the expression of V. cholerae virulence factors in response to host colonization (33). Here, we have tested this prediction using the zebrafish larva colonization model (9). For all genes examined, differential expression between planktonic and intestinal populations required CRP. We have paid particularly close attention to genes encoding the RTX toxin export machinery. Our data are consistent with repression of *rtxBDE* by CRP that is relieved within the intestinal tract and other nutrient-rich environments. Previous host colonization studies support our model. In particular, Mandlik and colleagues previously monitored global transcription in V. cholerae using RNA-seq (45). Their data demonstrate induction of *rtxBDE* within the intestinal tracts of both mice and rabbits. Furthermore, the same study detected repression of *rtxBDE* in M9 minimal medium compared to its expression in LB broth. Similarly, Boardman et al. noted repression of *rtxBDE* in nutrient-poor environments (46). We argue that CRP mediates these effects directly by binding sites upstream from the *rtxBDE* promoters (Fig. 2 to 4). Curiously, while the RTX toxin export machinery is expressed only upon nutrient upshift, the divergent *rtxHCA* genes that encode the RTX toxin appear constitutively transcribed (Fig. 2). Indeed, posttranscriptional control by the VqmR small RNA (sRNA) has previously been shown to regulate RTX toxin expression (45). We speculate that this allows the system to exist in a poised state so that toxin export is only triggered within a host organism.

Our data show that CRP is required for control of V. cholerae genes in addition to the *rtxBDE* operon in response to the intestinal environment (Fig. 5). Hence, CRP is integral to the regulatory network that controls V. cholerae lifestyle switching. This is intriguing, given that oral rehydration therapies (ORT) used to treat the effects of cholera contain glucose (26, 47). Hence, it is possible that ORT modification could be used to modulate the ability of V. cholerae to colonize a host and cause disease (26, 47). Complete dissection of the role CRP plays during V. cholerae lifestyle switching should provide an evidence base for any such ORT modification. In this regard, our findings provide an important starting point for further studies.

## MATERIALS AND METHODS

### Strains, plasmids, and oligonucleotides.

V. cholerae strains N16961 and E7946 are described by Heidelberg et al. and Miller et al., respectively (39, 48). The Δ*crp* derivative of E7946 was constructed by MuGENT using PCR oligonucleotides listed in Table S1 in the supplemental material (49). The E. coli K-12 strains JCB387 and DH5α are described by Page et al. (50) and Taylor et al. (51) and were used for general cloning and conjugation, respectively. Plasmid pRW50T was constructed by excision of the DNA fragment comprising *cynX* from pRW50 (52) using NheI and BstEII. The *oriT* region was amplified from plasmid RK2 (53) in such a way that *oriT* was flanked by NheI and BstEII restriction sites to facilitate ligation at the locus from which *cynX* was excised. Plasmid pDCRP-Vc is a derivative of pDCRP (54) that encodes the CRP protein of V. cholerae rather than that of E. coli. More-detailed descriptions of strains and plasmids, along with sequences of oligonucleotides, are provided in Table S1.

10.1128/mBio.00966-18.3 TABLE S1  Strains, plasmids, and oligonucleotides. Download TABLE S1, DOCX file, 0.03 MB. Copyright © 2018 Manneh-Roussel et al. 2018 Manneh-Roussel et al. This content is distributed under the terms of the Creative Commons Attribution 4.0 International license.

### ChIP and DNA sequencing.

Immunoprecipitations with monoclonal anti-CRP and anti-σ^70^ antibodies (Neoclone, Madison, WI) were done as described by Haycocks et al. (26) using lysates of strain N16961 (39). Lysates were prepared from mid-log-phase cells cultured in M9 minimal medium supplemented with 1% (wt/vol) fructose. Libraries were prepared using immunoprecipitated protein-DNA complexes immobilized with protein A-Sepharose. DNA fragments were then given blunt ends, poly(A) tails, and bar codes. This was done using an NEB quick blunting and ligation kit, the Klenow fragment (5′-3′ exo-; NEB), and NEXTflex chromatin immunoprecipitation-DNA sequencing (ChIP-seq) barcodes (Bioo Scientific). Following elution of complexes from the protein-A Sepharose, cross-links were reversed, and bar-coded libraries were amplified by PCR. The number of PCR cycles was determined empirically for each library. After amplification, the library concentration was quantified using Qubit analysis and real-time PCR. Equimolar library concentrations were pooled and sequenced using an Illumina MiSeq instrument.

### Bioinformatics.

The Fastq files obtained after DNA sequencing were converted into Fastq Sanger format, using FastqGroomer, and aligned to GenBank reference sequences (accession numbers AE003852.1 and AE003853.1) using BWA (Burroughs-Wheeler Aligner) for Illumina. The reference sequences correspond to chromosome I and chromosome II, respectively, of V. cholerae strain N16961. The resulting SAM (Sequence Alignment Map) files were converted to BAM (Binary Alignment Map) format using SAM-to-BAM. For each experiment, coverage per base was determined using multiBamSummary. Subsequent processing was done using R. Data were normalized to the same average read depth, and mean coverage per base was determined for each pair of biological replicates. Signals due to nonspecifically immunoprecipitated DNA present in a mock experiment were subtracted from the final binding profiles. To select peaks for CRP or σ^70^ binding, we used Artemis to generate a coverage plot and selected peaks. The peak centers were set as the center of the region passing the cutoff rounded to the nearest integer. Peaks for CRP and σ^70^ were defined as overlapping if the peak centers were within 250 bp of each other.

### Proteins.

The V. cholerae CRP protein was expressed in E. coli strain M182Δcrp and purified using cAMP-agarose as described previously (54). The V. cholerae RNA polymerase was purified using a method derived from that of Burgess and Jendrisak (55). Briefly, V. cholerae strain N16961 was grown to mid-log phase in 8 liters of LB medium. Cells were harvested by centrifugation and resuspended in 100 ml of lysis buffer (50 mM Tris-HCl, pH 7.5, 150 mM NaCl, 2 mM MgCl_2_, 0.1 mM dithiothreitol [DTT], 2 mM EDTA, 1 mM 2-mercaptoethanol, 5% glycerol, 0.2% Triton X-100, and 0.25 mg/ml lysozyme). One protease inhibitor cocktail tablet (Roche) was added per 20 ml of buffer. Cell lysis and DNA shearing were done using four 30-s pulses, at 20% output, with a Misonix, Inc., XL2020 tip sonicator. Lysates were cleared by centrifugation at 39,000 × *g* for 45 min at 4°C. Following filtration (0.45-µm filter), polymin P and ammonium sulfate precipitations were done as described in Burgess and Jendrisak (55). Precipitated protein was resuspended in TGED buffer (10 mM Tris-HCl, pH 7.9, 5% glycerol, 0.1 mM EDTA, and 0.1 mM DTT) containing 100 mM NaCl and passed through a HiPrep heparin FF column (GE Healthcare). The column was washed with 0.1 M NaCl TGED, and RNA polymerase was eluted in TGED using a gradient to 1 M NaCl. RNA polymerase-containing fractions were pooled and protein precipitated using ammonium sulfate. After resuspension in TGED, RNA polymerase was further purified using a Mono Q HR column (GE Healthcare). Column washing and protein elution were as described in the previous step. RNA polymerase-containing fractions were pooled and dialyzed against −80°C storage buffer (TGED, 0.1 M NaCl, 50% glycerol).

### DNase I footprinting and *in vitro* transcription.

For electrophoretic mobility shift assay (EMSA) experiments, DNA fragments were prepared using PCR as described by Shimada et al. (56), with oligonucleotides listed in Table S1. Protein binding and subsequent electrophoresis were done as described by Chintakayala et al. (57). For footprinting experiments, DNA fragments were prepared as described by Grainger et al. (58). Protein binding, DNA digestion, and electrophoresis were done as described by Singh and Grainger (59). Briefly, DNA fragments were labeled at one end using [γ-^32^P]ATP and T4 polynucleotide kinase and used at a final concentration of ~10 nM in footprinting reactions. All reaction mixtures contained an excess of herring sperm DNA (12.5 µg ml^−1^) as a nonspecific competitor. Our *in vitro* transcription assays were done as described by Haycocks et al. (26). DNase I-digested DNA and *in vitro*-generated RNA transcripts were analyzed on 6% DNA sequencing gels (Molecular Dynamics). The results were visualized using a Fuji phosphor screen and Bio-Rad Molecular Imager FX. Raw gel images are in Fig. S1d.

### Primer extension assays.

Transcript start sites were mapped by primer extension, as described previously (59), using RNA purified from a V. cholerae strain carrying the appropriately oriented *rtxBDE-rtxHCA* intergenic region cloned in pRW50T. The 5′-end-labeled primer D49724, which anneals downstream from the HindIII site in pRW50, was used in all experiments. Primer extension products were analyzed on denaturing 6% polyacrylamide gels calibrated with size standards derived from M13mp18 phage DNA sequencing reactions. Gels were visualized using a Fuji phosphor screen and Bio-Rad Molecular Imager FX.

### β-Galactosidase assays.

β-Galactosidase assays using lysates of liquid V. cholerae cultures were done as described previously (26) following the protocol of Miller (60). For experiments with zebrafish larva, colonization by V. cholerae was first instigated as described below. E3 medium was prepared as a 1 liter 50× stock containing 14.6 g NaCl, 0.65 g KCl, 2.20 g CaCl_2_, 4.05 g MgSO_4_, and 23.85 g HEPES adjusted to pH 7. A 1× dilution was prepared using ddH2O. Following infection, the larvae were euthanized with 2.5 mg/ml tricaine and the E3 medium was agitated to resuspended bacteria that had sunk to the bottom of the well. The V. cholerae-containing E3 medium was then transferred to a sterile bijou and the larvae to a sterile 1.5-ml dolphin microcentrifuge tube. Fish were washed with E3 medium by gentle pipetting to remove residual bacteria. Larvae were then homogenized to release bacterial cells using a hand-held motorized homogenizer, and E3 was added so that the homogenate had a volume similar to that of the isolated medium. Two drops each of toluene and 1% (wt/vol) sodium deoxycholate were added to each sample, and the resulting cell lysates were assayed for β-galactosidase activity. To normalize for cell numbers, 0.5 µl of each cell suspension was diluted in 1.5 ml of E3 medium prior to cell lysis. One hundred microliters of this suspension was spread on LB agar plates containing 5 µg/ml tetracycline, 100 µg/ml streptomycin, 50 µg/ml spectinomycin, and 40 µg/ml X-Gal (5-bromo-4-chloro-3-indolyl-β-d-galactopyranoside). This allowed for confident selection of V. cholerae cells containing pRW50T derivatives that were enumerated by counting the number of colonies formed after overnight incubation at 37°C. All assay values are the means of the results of three independent experiments with a standard deviation equivalent to <10% of the mean β-galactosidase activity.

### Zebrafish larva colonization and survival assays.

Adult zebrafish were kept at pH 7.5 and 26°C in a recirculating tank system, with light/dark cycles of 14/10 h, at the University of Birmingham aquatic facility. Zebrafish care, breeding, and experimentation were done according to the Animal (Scientific Procedures) Act 1986 (61) under home office project license 40/3681. Zebrafish embryos derived from the wild-type AB strain (62) were harvested in petri dishes containing water from the fish system. After harvesting, between 50 and 60 embryos were transferred to 90-mm petri dishes containing 25 ml of E3 medium supplemented with 0.03% (vol/vol) methylene blue and 0.02 mg/ml 1-phenyl 2-thiourea. The embryos were incubated at 32°C for 4 days with light/dark cycles of 14/10 h. The incubation medium was regularly replaced to minimize microbial contamination. On day 3 of the incubation, required strains of V. cholerae were streaked to generate single colonies that were used to inoculate 5 ml of M9 minimal medium. The resulting cultures were incubated overnight at 37°C with shaking. One milliliter of the overnight culture was transferred to 5 ml of fresh M9 minimal medium the following day. The resulting culture was incubated at 37°C with shaking until mid-log phase. Cells were then harvested by centrifugation and washed three times with 5 ml of E3 buffer by sequential resuspension and centrifugation. After washing, the cells were resuspended in 5 ml of E3 medium, the optical density was determined, and 10^6^ cells were transferred into each well of a 24-well cell culture plate. The larvae were sedated by adding 166 µl of 40-mg/ml tricaine to the petri dish. Five larvae were then transferred to each well of the culture plate, which was incubated at 30°C overnight. Death was determined by loss of movement and heartbeat in opaque larvae that had settled at the bottom of the well.

### Microscopy.

Zebrafish embryos colonized with V. cholerae were imaged using a Zeiss Axio Observer Z1 microscope with 10× objective for fluorescence and differential interference contrast. Prior to visualization, embryos were immobilized in 0.4% low-melting-point agarose in E3 buffer and 160 µg/ml tricaine. Imaging was done at 32°C and humidity maintained at 80% using an OkoLab stage. The ImageJ image processing package (NIH) software was used to visualize the images and merge the fields.

### Data availability.

DNA sequencing reads are stored in ArrayExpress under accession number E-MTAB-6472. Genome Browser files (Data Set S1 to S6) and instructions (Text S1) are provided in the supplemental material.

10.1128/mBio.00966-18.4 DATA SET S1  N16961 chromosome I GenBank file. Download DATA SET S1, TXT file, 6 MB. Copyright © 2018 Manneh-Roussel et al. 2018 Manneh-Roussel et al. This content is distributed under the terms of the Creative Commons Attribution 4.0 International license.

10.1128/mBio.00966-18.5 DATA SET S2  N16961 chromosome II GenBank file. Download DATA SET S2, TXT file, 2.2 MB. Copyright © 2018 Manneh-Roussel et al. 2018 Manneh-Roussel et al. This content is distributed under the terms of the Creative Commons Attribution 4.0 International license.

10.1128/mBio.00966-18.6 DATA SET S3  CRP ChIP-seq Artemis graph file for N16961 chromosome I. Download DATA SET S3, TXT file, 17.5 MB. Copyright © 2018 Manneh-Roussel et al. 2018 Manneh-Roussel et al. This content is distributed under the terms of the Creative Commons Attribution 4.0 International license.

10.1128/mBio.00966-18.7 DATA SET S4  CRP ChIP-seq Artemis graph file for N16961 chromosome II. Download DATA SET S4, TXT file, 6.4 MB. Copyright © 2018 Manneh-Roussel et al. 2018 Manneh-Roussel et al. This content is distributed under the terms of the Creative Commons Attribution 4.0 International license.

10.1128/mBio.00966-18.8 DATA SET S5  RNA polymerase σ^70^ ChIP-seq Artemis graph file for N16961 chromosome I. Download DATA SET S5, TXT file, 10.7 MB. Copyright © 2018 Manneh-Roussel et al. 2018 Manneh-Roussel et al. This content is distributed under the terms of the Creative Commons Attribution 4.0 International license.

10.1128/mBio.00966-18.9 DATA SET S6  RNA polymerase σ^70^ ChIP-seq Artemis graph file for N16961 chromosome II. Download DATA SET S6, TXT file, 4.2 MB. Copyright © 2018 Manneh-Roussel et al. 2018 Manneh-Roussel et al. This content is distributed under the terms of the Creative Commons Attribution 4.0 International license.

10.1128/mBio.00966-18.1 TEXT S1  Instructions for viewing ChIP-seq data in the Artemis genome browser. Download TEXT S1, PDF file, 0.6 MB. Copyright © 2018 Manneh-Roussel et al. 2018 Manneh-Roussel et al. This content is distributed under the terms of the Creative Commons Attribution 4.0 International license.
