# Supplementary material for: cAMP Receptor Protein Controls Vibrio cholerae Gene Expression in Response to Host Colonization
Source: mBio. 2018 Jul 10;9(4):e00966-18. doi: 10.1128/mBio.00966-18 (PMC6050953; doi:10.1128/mBio.00966-18)
Supplement: FIG S1 [file mbo004183969sf1.pdf]

Figure S1a

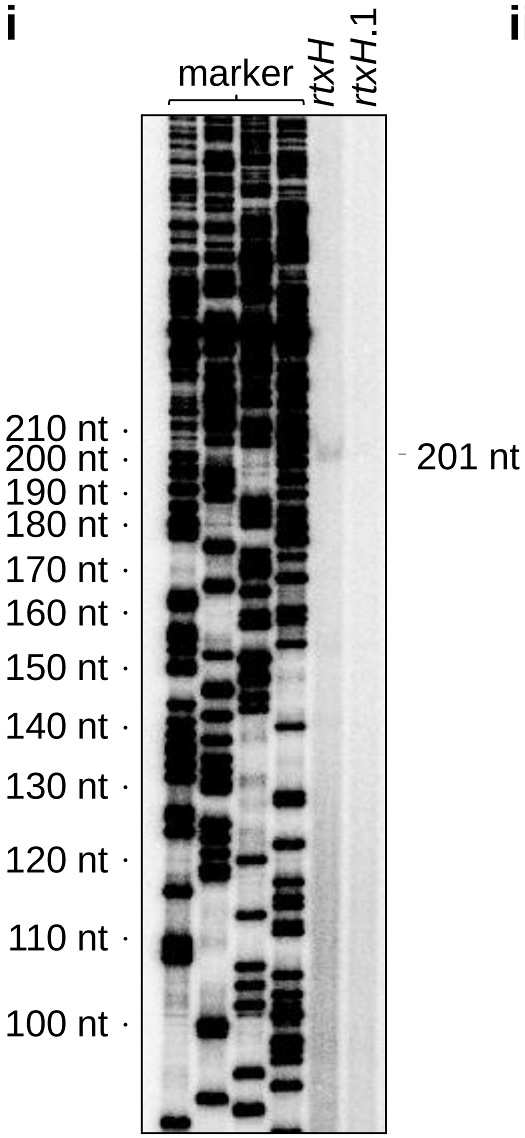

ii

rtxH

```
CACCGCTTGTTAACATGTTCAATCCTCAATCACCGTCAGCGT

TGTATGATAAATGTTGAAATAGTCTATTTTGTAGTCAATTTT

GATACAAATTAATGATATGAGTCACGTTAATGTGCAATTTGT
                    CRP1                    CRP2
CTGATGGTTGCATCTGTGTGAAATTGAGGGAATACCGCACCA

TTAAATTAGAATATATACTCTAATTTAATGTGTTTAAGTCGG
                                         PrtxH
TTGATAGCCATGGTTTAACGATGTAATATCCCGCCTTAAAAT

AAATAATACAATAATAACCCTAGGTTTGATTTGTTATATTTT

TGCCCTTTGGGCTTGCTGGGAAAGATTGGTTCATAGAAACCA

TCTTTTCATATCTGTGTAAAATAAATG
```

rtxH.1

```
CACCGCTTGTTAACATGTTCAATCCTCAATCACCGTCAGCGT

TGTATGATAAATGTTGAAATAGTCTATTTTGTAGTCAATTTT

GATACAAATTAATGATATGAGTCACGTTAATGTGCAATTTGT
                    CRP1                    CRP2
CTGATGGTTGCATCTGTGTGAAATTGAGGGAATACCGCACCA

TTAAATTAGAATATATACTCTAATTTAATGTGTTTAAGTCGG

TTGATAGCCATGGTTTAAC
```

Figure S1a: Transcription from P*rtxH* requires identified promoter elements. Panel i) shows the results of primer extension assays that detect the rtxH transcript derived from plasmid pRW50T carrying the *rtxH* (Panel ii, top) or *rtxH*.1 (Panel ii, bottom) DNA fragment. In Panel ii) P*rtxH* is highlighted blue and CRP binding sites are shown in orange.

Figure S1b

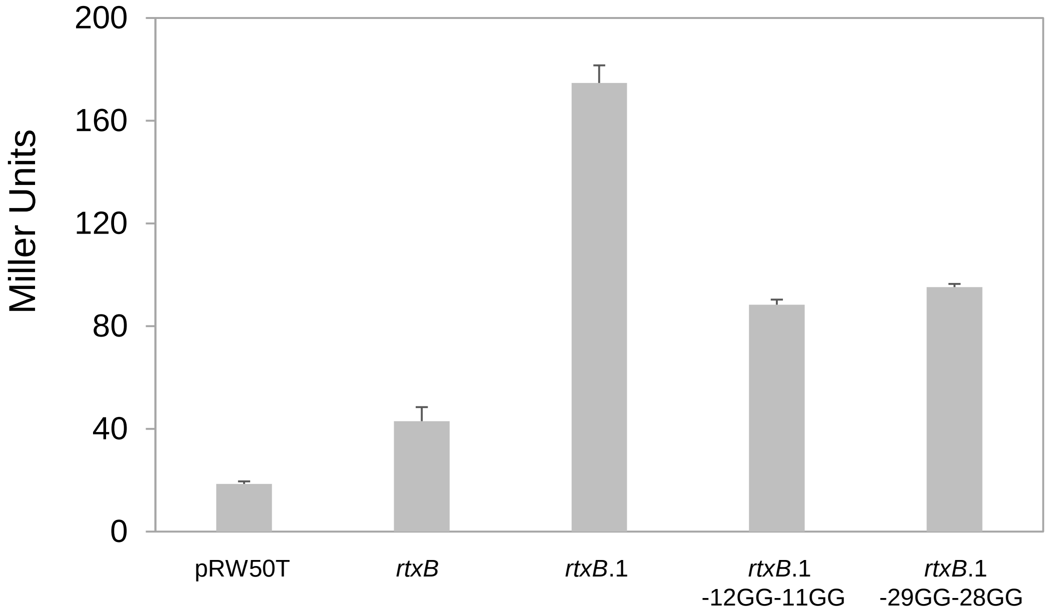

Figure S1b: P1rtxB and P2rtxB make similar contributions to *rtxB* transcription. The figure shows β-galactosidase activity measurements for lysates of *V. cholerae* cells transformed with pRW50T derivatives carrying the full length *rtxB* regulatory region (*rtxB*) a truncated derivative lacking CRP binding sites (*rtxB.1*) or versions of the truncated fragment with indicated promoter mutations.

Figure S1c

i

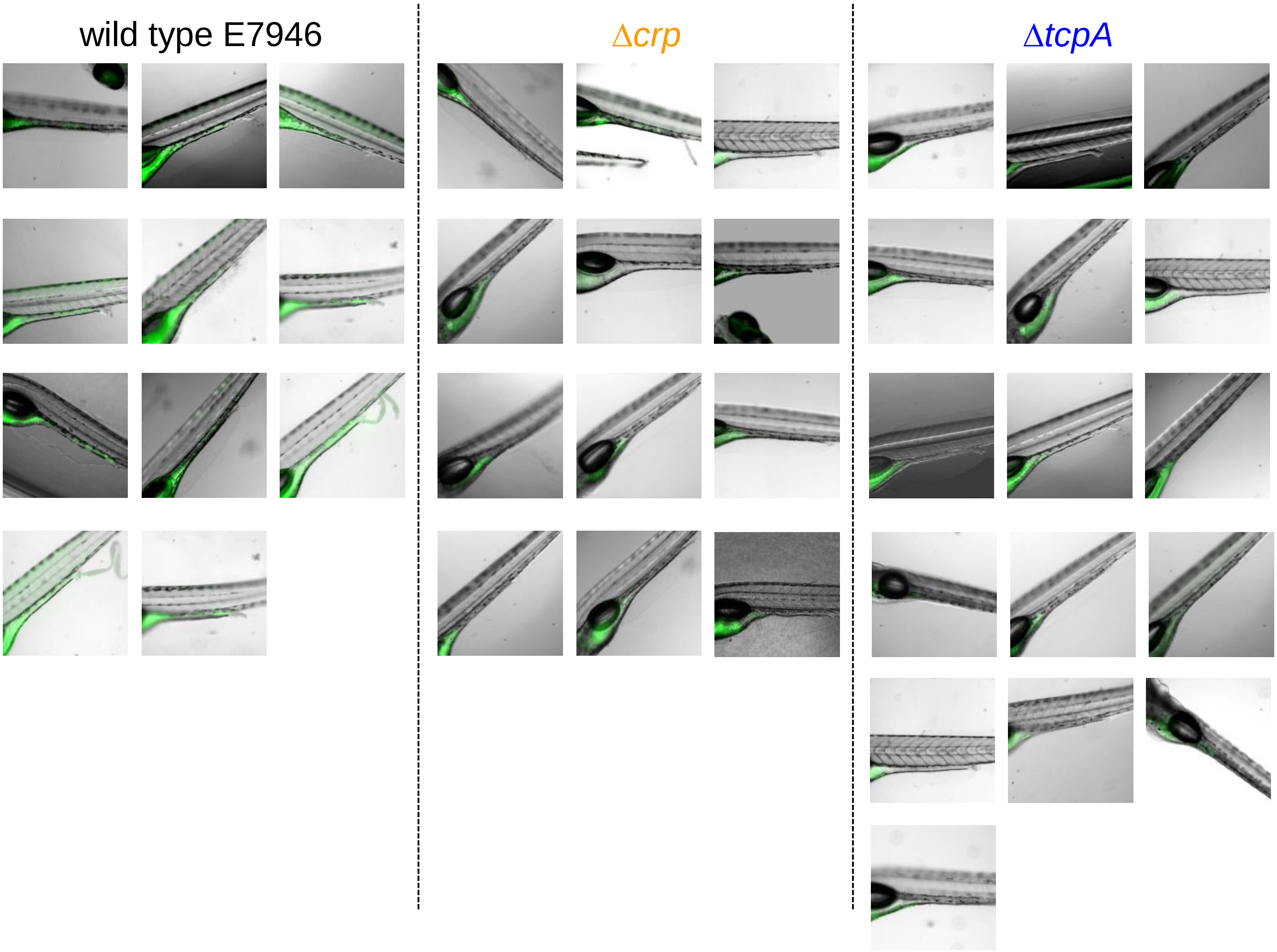

ii

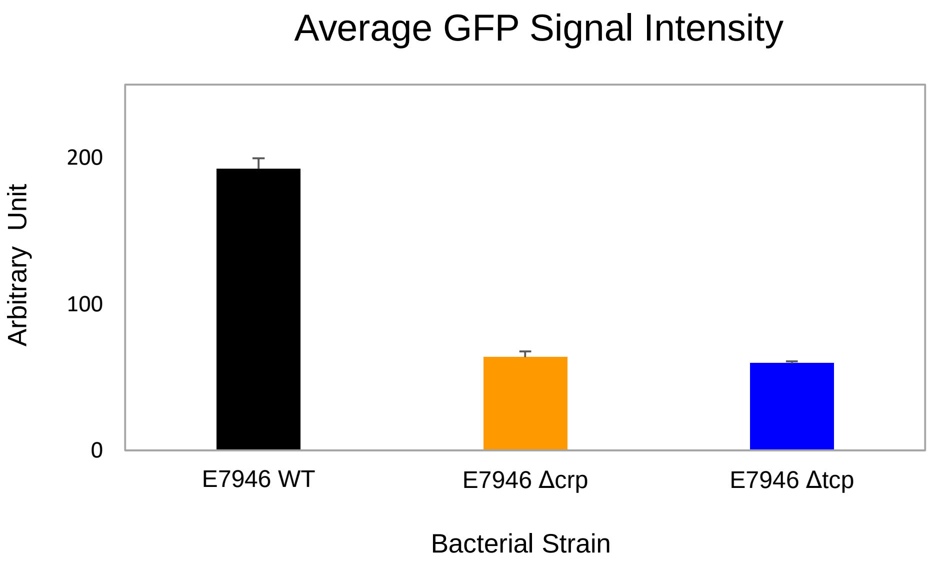

Figure S1c: Effects of *crp* and *tcpA* on zebrafish larvae colonisation. Panel i) shows images from multiple zebrafish larvae colonised with the indicated *V. cholerae* strain. All strains were transformed with plasmid pMW-GFP to facilitate visualisation of bacteria. Panel ii) shows quantified flouresence from multiple microscopy images.

Figure S1d

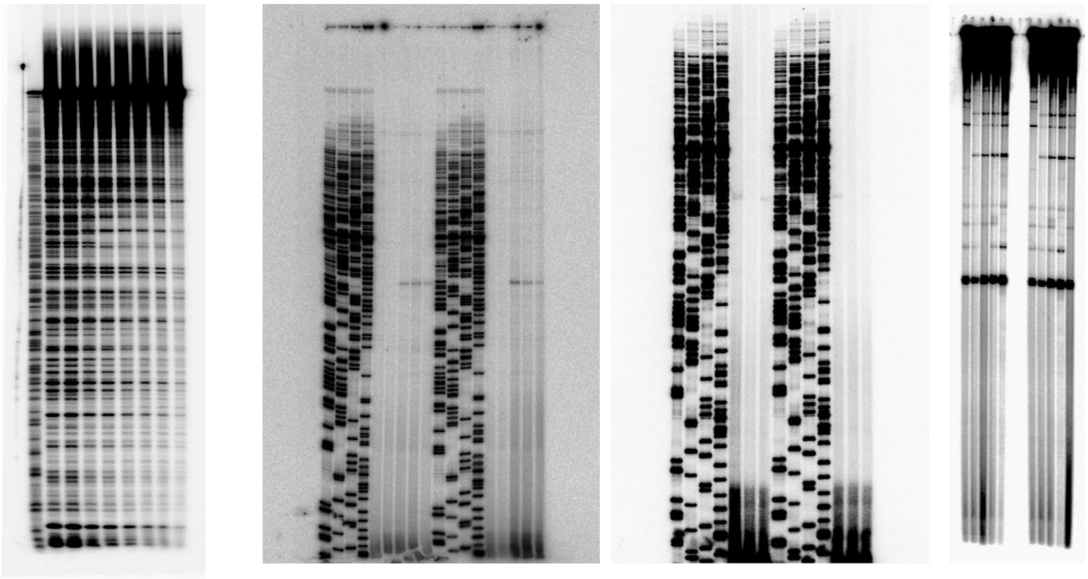

Figure S1d: Raw gel images.
