# Supplementary material for: cAMP Receptor Protein Controls Vibrio cholerae Gene Expression in Response to Host Colonization
Source: mBio. 2018 Jul 10;9(4):e00966-18. doi: 10.1128/mBio.00966-18 (PMC6050953; doi:10.1128/mBio.00966-18)
Supplement: TABLE S1 [file mbo004183969st1.docx]

**Table S1: Strains, plasmids and oligonucleotides**

**Name Sequence 5'-3' Description**

*Oligonucleotide primers used to generate promoter DNA fragments by PCR*

*rtxB* F GGCTGCGAATTCATTCTAATTTAATGGTGCGGTATTCCC Forward primer used to amplify *rtxB* fragment

*rtxB* R GCCCGAAGCTTCACCGCTTGTTAACATGTTCA Reverse primer used to amplify *rtxB* fragment

*rtxH* F GGCTGCGAATTCCACCGCTTGTTAACATGTTCAATCC Forward primer used to amplify *rtxH* fragment

*rtxH* R GCCCGAAGCTTCATTCGTACCTCCTTTTATTTTAAGGCGGG Reverse primer used to amplify *rtxH* fragment

ATATTACATCGTTAAACCATGGCTATCAACCGACTTAAACAC

*tolC* F GGCTGCGAATTCTGCCGACTACCAAGTGATGGCT Forward primer used to amplify *tolC* fragment

*tolC* R GCCCGAAGCTT CATCGGTCCTATTCCTGACGTG Reverse primer used to amplify *tolC* fragment

*nudF* F GGCTGCGAATTCCATCGGTCCTATTCCTGACGTG Forward primer to amplify *nudF* fragment

*nudF* R GCCCGAAGCTTCATTCGTACCTCCTATTGATGAAC Reverse primer to amplify *nudF* fragment

*acfD* F GGCTGCGAATTCATAACTTGAATAGTTGATTTCA Forward primer to amplify *acfD* fragment

*acfD* R GCCCGAAGCTTCATCTTAAGCCTATTAAACAAAAAACAAGA Reverse primer used to amplify *acfD* fragment

ATTAATTATCCTTG

*acfA* F GGCTGCGAATTCCATCTTAAGCCTATTAAACAAAAAACAAG Forward primer to amplify *acfA* fragment

*acfA* R GCCCGAAGCTTCATTTTTTACTCCTATTTTTACCTGTG Reverse primer used to amplify acfA fragment

*rtx*.1 F GGCTGCGAATTCTTAATTTGTATCAAAATTGAC Forward primer used to generate truncated

*rtxB*.1 fragment lacking *crp* sites

*rtx*.1 P2 F GGCTGCGAATTCTTAATTTGTATCAAAATTGACTACAAAAT Forward primer used to change bases -11 and

AGACTATTTCAACATTGGTCATACAACGCTGACG -12 in the *rtxB*.1 fragment

*rtx*.1 P4 F GGCTGCGAATTCTTAATTTGTATCAAAATTGACTACAAAAG Forward primer used to change bases -28 and

GGAC -29 in truncated *rtxB*.1 fragment

CRP3.2.4 R GCCCGAAGCTTCACCGCTTGTTAACATGTTCAATCCTCAATC Reverse primer used to change bases

ACCGTCAGCGTTGTATGACCAATGTTGAAATAGTCCCTTTTG -11, -12 and -28, -29 in the *rtxB*.1.

TAGTC

*Oligonucleotide primers used for construction and Sanger sequencing of plasmid DNA constructs*

pRW50 F GTTCTCGCAAGGACGAGAATTTC Anneals upstream of pRW50T *EcoR*I site

pRW50 R AATCTTCACGCTTGAGATAC Anneals downstream of pRW50T *Hin*dIII site

pSR F GCATTTATCAGGGTTATTGTCTC Anneals upstream of *EcoR*I site in pSR

pSR R CATCACCGAAACGCGCGAGG Anneals downstream of *Hin*dIII site in pSR

*xylE* mid F TCATAGGTCGCCGGCAATTCG Anneals within *xylE* gene of pRWXT

*Oligonucleotide used to generate DNA fragments for use in MuGENT experiments*

Arm1 Δ*crp* F1 CGCCAAACCAAGATTAACACTG Generation of arm 1 of Δ*crp* construct

Arm1 Δ*crp* R1 ggttatcggggcacttagcgagtgcCCATAATAATCTCACT Generation of arm 1 of Δ*crp* construct

TCCTCTGC

Arm2 Δ*crp* F2 gcagaggaagtgagattattatggGCACTCGCTAAGTGCC Generation of arm 2 of Δ*crp* construct

CCGATAACC

Arm2 Δ*crp* R2 GCTGAGATGCGCGGCGTTTCCGATGC Generate arm 2 of Δ*crp* construct

Δ*crp* MASC F GCACCCACATTAACTTTCAGCGTGTTCC Forward primer used to determine

presence or absence of *crp* site

Δ*crp* MASC R GCTGAAAGTGGAAGAGTGTTCACC Reverse primer used to determine presence or

absence of crp site

VC1807::kan F GTAGAATAAGTGCGGCGTTGAGCC Forward primer used to amplify kan gene

inserted into VC1807

VC1807::kan R GCGCCCAATGTTGTCCCTTTGATG Reverse primer used to amplify kan gene

inserted into VC1807

oriT F ATTCGCGAATTCGGTAACCCCCGTTGAGCACCGCCAGGTG Forward primer used to amplify oriT, *traJ* and

*traK* genes from Birmingham IncP alpha plasmid, RK2

oriT R ATTCGCAAGCTTGCTAGCCTTTTCCGCTGCATAACCCTGC Reverse primer used to amplify oriT, *traJ* and

*traK* genes from Birmingham IncP alpha plasmid, RK2

pRWXT F GCCTTGAGTCCACGCTGGATCCCCGACGACGACATGGC Forward primer used to amplify *xylE* gene

from pRWX plasmid

pRWXT R GCCCGGAATTCGACAACATGAACTATGAAGAGG Reverse primer used to amplify *xylE* gene

from pRWX plasmid

T7 primer GTAAAACGACGGCCAGT Universal primer for T7 sequencing reactions

D49724 GGTTGGACGCCCGGCATAGTTTTTCAGCAGGTCGTTG

*Bacterial Strains*

DH5α F– *endA1*, *glnV44*, *thi-1*, *recA1*, *relA1*, *gyrA96*, *deoR*, *nupG*, Grant *et al*., 1990

*purB20*, φ80d*lacZ*ΔM15, Δ(*lacZYA-argF*) U169, *hsdR17*

(rK–mK+), λ–

DH5α (pRK2013) DH5α, KanR, oriColE1, RK2- Mob+ Tra+ Connell *et al*., 1995

JCB387 Δ*nirB* Δ*lac* Page *et al*. 1990

N16961 Wild-type Inaba El Tor, SmR, HapR^-^  Heidelberg *et al*., 2000

E7646 Wild type SmR derivative of E7946, El Tor Ogawa Dalia *et al*., 2014

E7946 Δ*crp* Derivative of E7946 WT, VC1807::Kan, Δ*crp*  This work

*Plasmids*

pRW50 16.9 kb plasmid with *Eco*RI-*Hin*dIII cloning site upstream Lodge *et al*., 1992

of *lacZ*. Encodes Tet^R^ and has RK2 origin.

pRW50T Derivative of pRW50, Tra+ This work

pSR 2.6 kb A pBR322 derived plasmid with *Eco*RI-*Hin*dIII cloning Kolb *et al*., 1995a

site upstream of a λ*oop* transcriptional terminator.

Encodes Amp^R^ and has a ColE1 origin
