# Supplementary material for: cAMP Receptor Protein Controls Vibrio cholerae Gene Expression in Response to Host Colonization
Source: mBio. 2018 Jul 10;9(4):e00966-18. doi: 10.1128/mBio.00966-18 (PMC6050953; doi:10.1128/mBio.00966-18)

1. Download the Artemis genome browser (https://www.sanger.ac.uk/science/tools/artemis) or launch directly from the website.

2. Open Genbank file for the desired *V. cholerae* chromosome (supplementary data set S1 or S2) in Artemis.

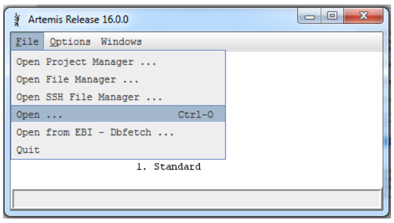

3. Under the “graph” tab select “Add User Plot” and open one of the ChIP-seq data sets (supplementary data sets S3-S6).

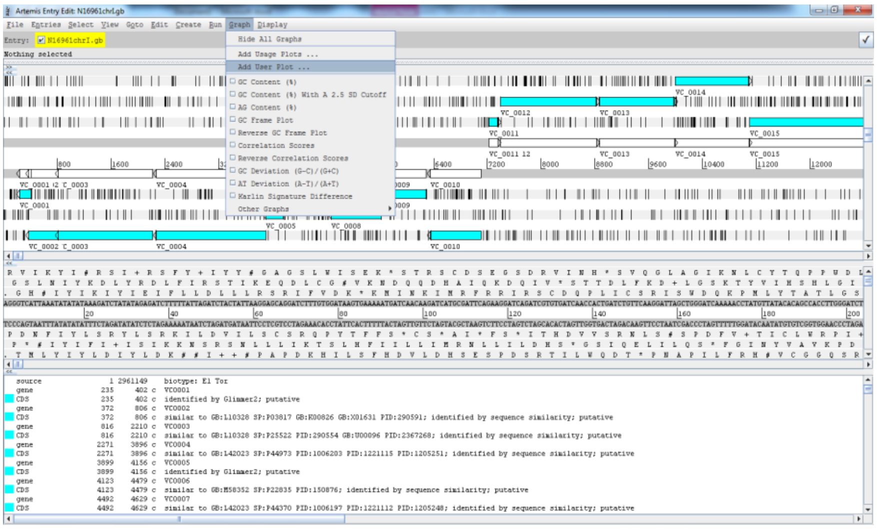

4. The ChIP-seq data will appear as a graph above the genome annotation. Add further data as required by importing additional user plots.

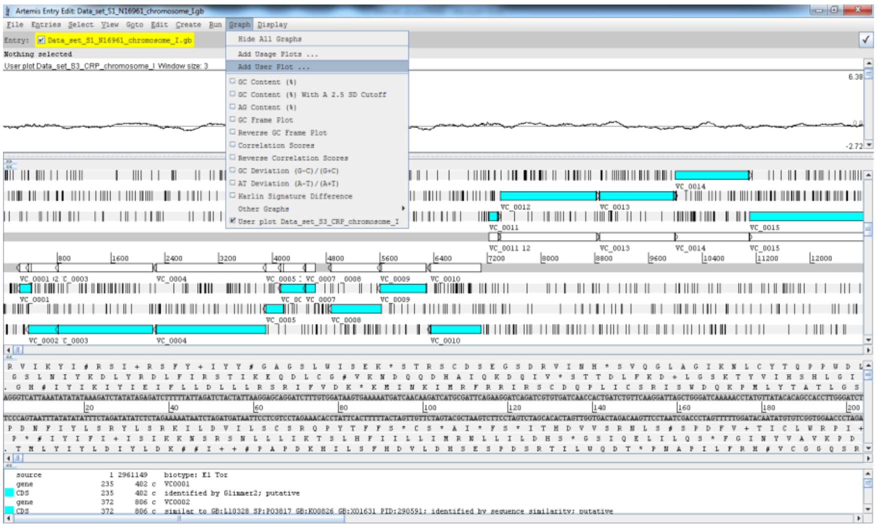

5. Right click in the graph window to alter scale, colour, window size and so on.

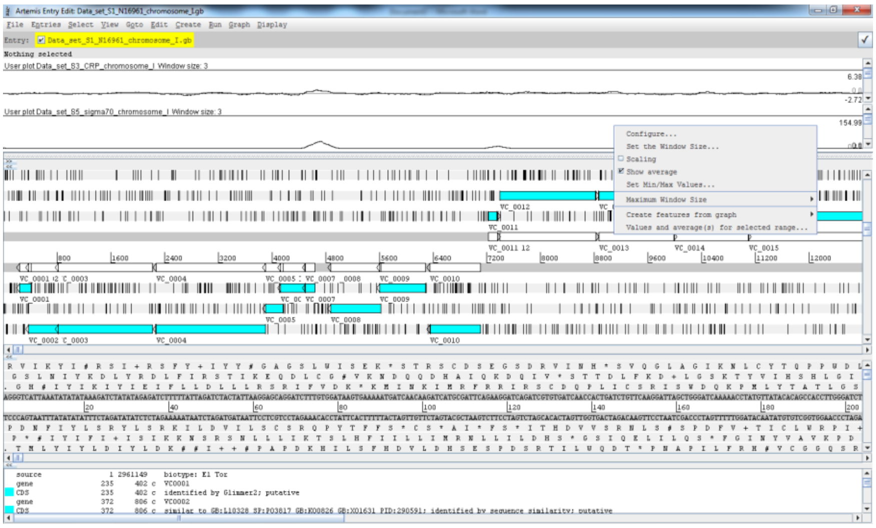

6. Browse the data to find features of interest.

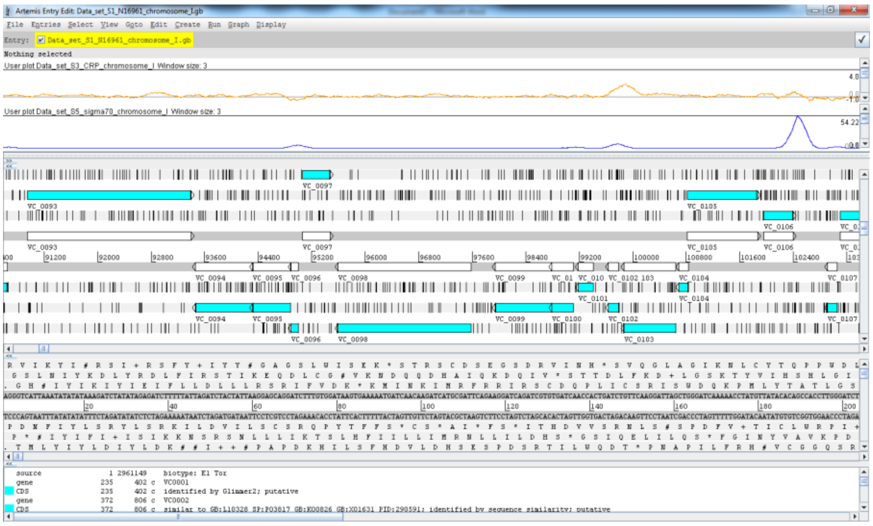

Supplement: TEXT S1 [file mbo004183969s1.pdf]
